# Editorial: Educational strategies and multimodal treatments for effective management of temporomandibular disorders and orofacial pain

**DOI:** 10.3389/fresc.2026.2011567

**Published:** 2026-10-23

**Authors:** Eleuterio A. Sánchez-Romero, Rosana Cid-Verdejo, Rob Sillevis, Juan Nicolás Cuenca-Zaldívar

**Affiliations:** 1 Facultad de Ciencias Biomédicas y de la Salud, Universidad Alfonso X el Sabio (UAX), Madrid, Spain; 2 Department of Rehabilitation Sciences, Florida Gulf Coast University, Fort Myers, FL, United States; 3 Research Group in Nursing and Health Care, Puerta de Hierro Health Research Institute-Segovia de Arana (IDIPHISA), Majadahonda, Spain; 4 Interdisciplinary Research Group on Musculoskeletal Disorders, Madrid, Spain; 5 Physiotherapy and Orofacial Pain Working Group, Sociedad Española de Disfunción Craneomandibular y Dolor Orofacial (SEDCYDO), Madrid, Spain; 6 Faculty of Dentistry, Universidad Complutense de Madrid, Madrid, Spain; 7 Department of Clinical Dentistry, Faculty of Biomedical Sciences, Universidad Europea de Madrid, Madrid, Spain; 8 Physical Therapy Unit, Primary Health Care Center El Abajón, Las Rozas de Madrid, Spain

**Keywords:** differential diagnosis, evidence-based practice, interdisciplinary care, multimodal care, orofacial pain (OFP), patient education, physiotherapy, temporomandibular disorders

To properly manage temporomandibular disorders (TMDs) and orofacial pain, it is necessary to go beyond simply choosing from the available treatments. Clinical decisions must consider an accurate diagnosis, the individual patient's presentation and priorities, clearly established therapeutic objectives, and the strength of the available evidence. The contributions in this Research Topic span clinical observation, evidence synthesis, technological feasibility, pilot interventions, and research mapping. Together, they pose a key question: how can educational and multimodal approaches be combined into coherent, individualized, evidence-based treatment strategies? The 2026 AADOCR position statement offers a timely framework by stressing conservative, reversible, and patient-centered management (1). Figure 1 summarizes the six conceptual components and cross-cutting themes addressed in this editorial.

**Figure 1:**
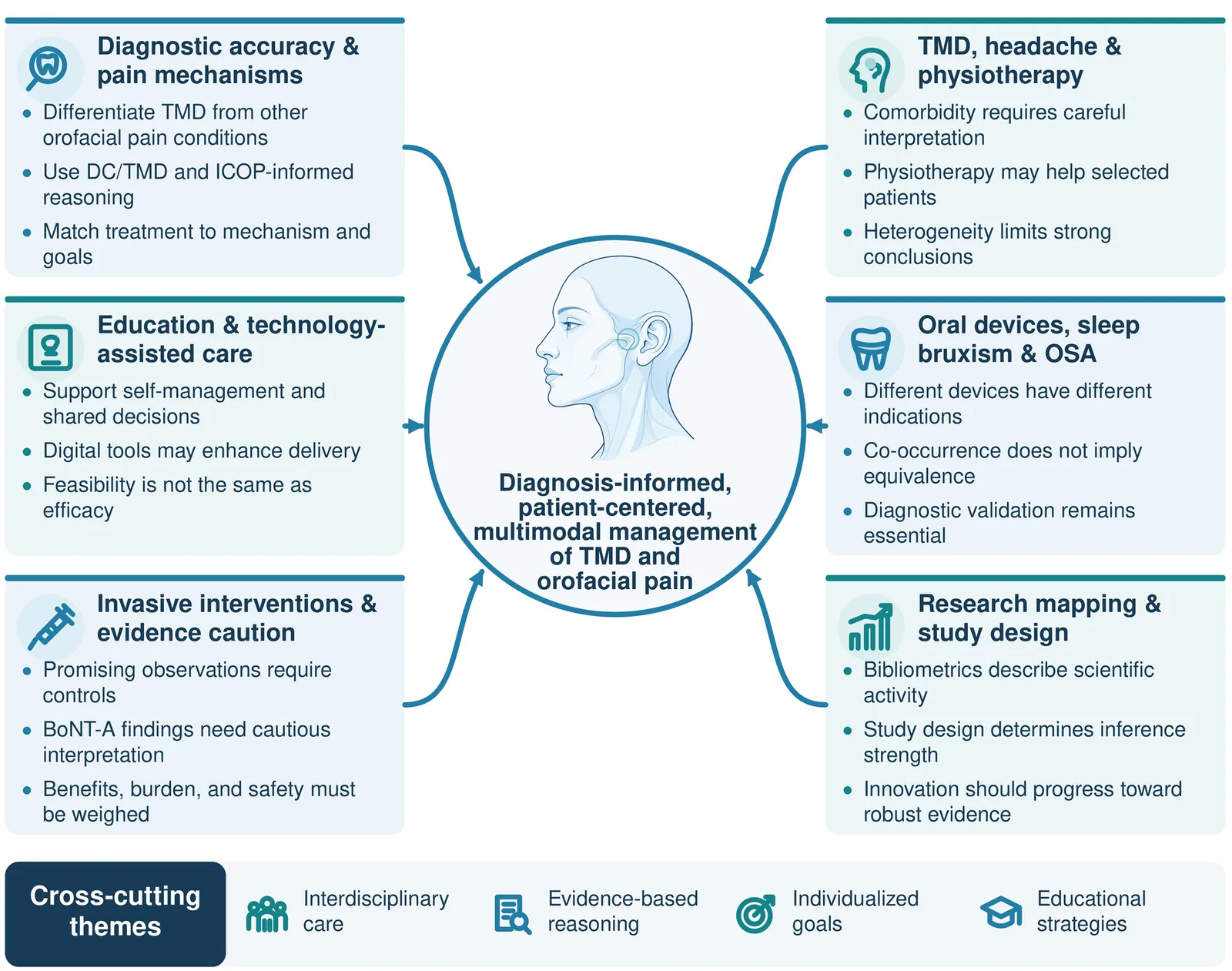
Conceptual components addressed in the editorial. Six conceptual components are organized around diagnosis-informed, patient-centered multimodal management of temporomandibular disorders (TMD) and orofacial pain. The lower band identifies cross-cutting themes: interdisciplinary care, evidence-based reasoning, individualized goals, and educational strategies. The central craniofacial profile is schematic. DC/TMD, diagnostic criteria for temporomandibular disorders; ICOP, International Classification of Orofacial Pain; OSA, obstructive sleep apnea; BoNT-A, botulinum toxin type A.

Accuracy in diagnosis must be the first step. TMDs do not constitute a single clinical condition, and not all craniofacial pain should be explained using a TMD approach. The DC/TMD integrate physical diagnoses with an evaluation of pain-related disability and psychosocial factors, whereas ICOP places TMDs within the wider range of possible causes of orofacial pain (2, 3). Li et al. demonstrate this distinction through a case of severe postherpetic neuralgia accompanied by peripheral facial paralysis treated with acupuncture and electroacupuncture as part of a multi-component intervention. Improvement in pain and facial nerve function was noted, yet this involves neuropathic orofacial pain and not TMD, and the combined intervention does not allow the improvement to be attributed to acupuncture alone. The report nevertheless highlights the wide diagnostic scope of orofacial pain and the need to differentiate clinical observation from evidence of treatment efficacy.

Diagnostic specificity is also important in relation to TMDs and headache. Quilghini et al. found five randomized controlled trials of physiotherapy in patients with chronic headache and TMD, with mixed results and substantial heterogeneity in quality, interventions, and outcomes. Their findings support further research in appropriately selected patients, but warn against interpreting the coexistence of TMD, cervical symptoms, and headache as proof of a single underlying disorder. The BMJ evidence review supports interventions aimed at promoting movement, functional ability, and coping strategies in chronic TMD pain (4), while the accompanying guideline strongly recommends supervised jaw exercises and stretching, postural exercises, certain manual techniques, cognitive behavioral therapy, and standard care including education and home exercise (5). Multimodal management should therefore involve deliberate integration, not simply accumulation, of treatment elements.

In this model, education is of great importance. Patient education should go beyond information on anatomy, pathology, or treatment and help patients understand their symptoms, participate in decisions, address unhelpful responses to pain, and build confidence in self-management. Clinical education is equally important: professionals must distinguish TMDs from other causes of orofacial pain, recognize diagnostic uncertainty and the limits of their competence, and know when referral or interdisciplinary assessment is required.

New technologies could offer innovative ways of delivering and reinforcing education. Conen et al. carried out two uncontrolled feasibility studies using augmented reality for psychoeducation and mindfulness-based body scanning in chronic low back pain. They reported high feasibility and positive user experience while stressing that controlled trials are needed before efficacy can be determined. Although these results cannot be directly applied to TMD, they suggest a possible avenue for orofacial pain research. Technology may alter how educational and behavioral interventions are delivered, but not the evidence required to demonstrate clinical effectiveness. Novelty, engagement, and patient satisfaction are valuable, but are not equivalent to effectiveness. Likewise, a recent network meta-analysis found no statistically significant differences in pain outcomes between biofeedback and its comparators (6), underscoring the need to measure the constructs an intervention is intended to change.

The interpretation of oral devices depends on their clinical purpose. Wang et al. examine sports mouthguards as protective devices intended to reduce dental and orofacial trauma. Their indication differs from stabilization or occlusal appliances used in TMD management and mandibular advancement devices used for obstructive sleep apnea (7). Sleep bruxism, obstructive sleep apnea, and TMD may coexist, but co-occurrence does not establish diagnostic equivalence or causality. These conditions should be assessed separately according to their respective diagnostic frameworks. Portable electromyographic assessment of sleep bruxism has very low-certainty evidence (8), reinforcing the need for diagnostic validation.

The contrast between promising clinical observations and evidence of causality is clearest for more invasive interventions. Macedo de Sousa et al. monitored 25 patients with myogenic TMD who received a single session of bilateral intramuscular botulinum toxin type A injections. Improvements were reported in pain, functional limitations, and oral-health-related quality of life, with no adverse effects over 12 weeks. Yet, without a control group, efficacy cannot be established because natural course, regression to the mean, expectations, concurrent care, and contextual factors cannot be distinguished from treatment effects. Likewise, the absence of adverse events in a small short-term cohort does not establish long-term safety. A multicenter trial found no additional pain relief with botulinum toxin versus saline over nine months (9). BMJ conditionally advises against botulinum toxin and reversible splints (5); British guidance permits selected splint use but considers botulinum toxin evidence uncertain (10). Interpretation depends on population, comparator, outcome, and care pathway.

Research volume must also be interpreted cautiously. Liao et al. found substantial growth in research on dry needling and pain, mapping themes in clinical effectiveness, trial design, outcome assessment, and risk management. Bibliometric analyses describe how a field has developed, but publication numbers, citations, and research clusters cannot establish therapeutic efficacy. Clinical claims must remain aligned with what each study design can establish: case reports generate hypotheses, feasibility studies examine acceptability and implementation, bibliometric studies map research activity, and controlled comparative trials provide more direct estimates of treatment effects.

Above all, these contributions point out the limitations of focusing on TMD and orofacial pain solely through individual treatment methods. The clinically important question is not whether physiotherapy, education, needling, an oral device, pharmacological intervention, or emerging technology is effective in all cases. Instead, treatment should be based on the pain condition, relevant functional impairments, psychosocial factors, patient priorities, and the therapeutic goal. A clinically useful multimodal approach begins with a question that precedes treatment selection: what needs to change for this particular patient? Diagnosis identifies the condition, but does not by itself define the whole plan of care. Assessment should identify modifiable biological, psychological, behavioral, and functional factors; education should establish shared understanding and promote self-management; and further interventions should be chosen according to explicit treatment targets. Multimodal care does not imply that all patients need several treatments. Rather, complementary interventions should be combined when each meets a specific clinical need and offers sufficient expected benefit to justify its burden, cost, and potential risk. Figure 2 illustrates this diagnosis-informed pathway from comprehensive assessment and patient-specific treatment targets to first-line management, selected adjunctive options, and outcome reassessment.

**Figure 2:**
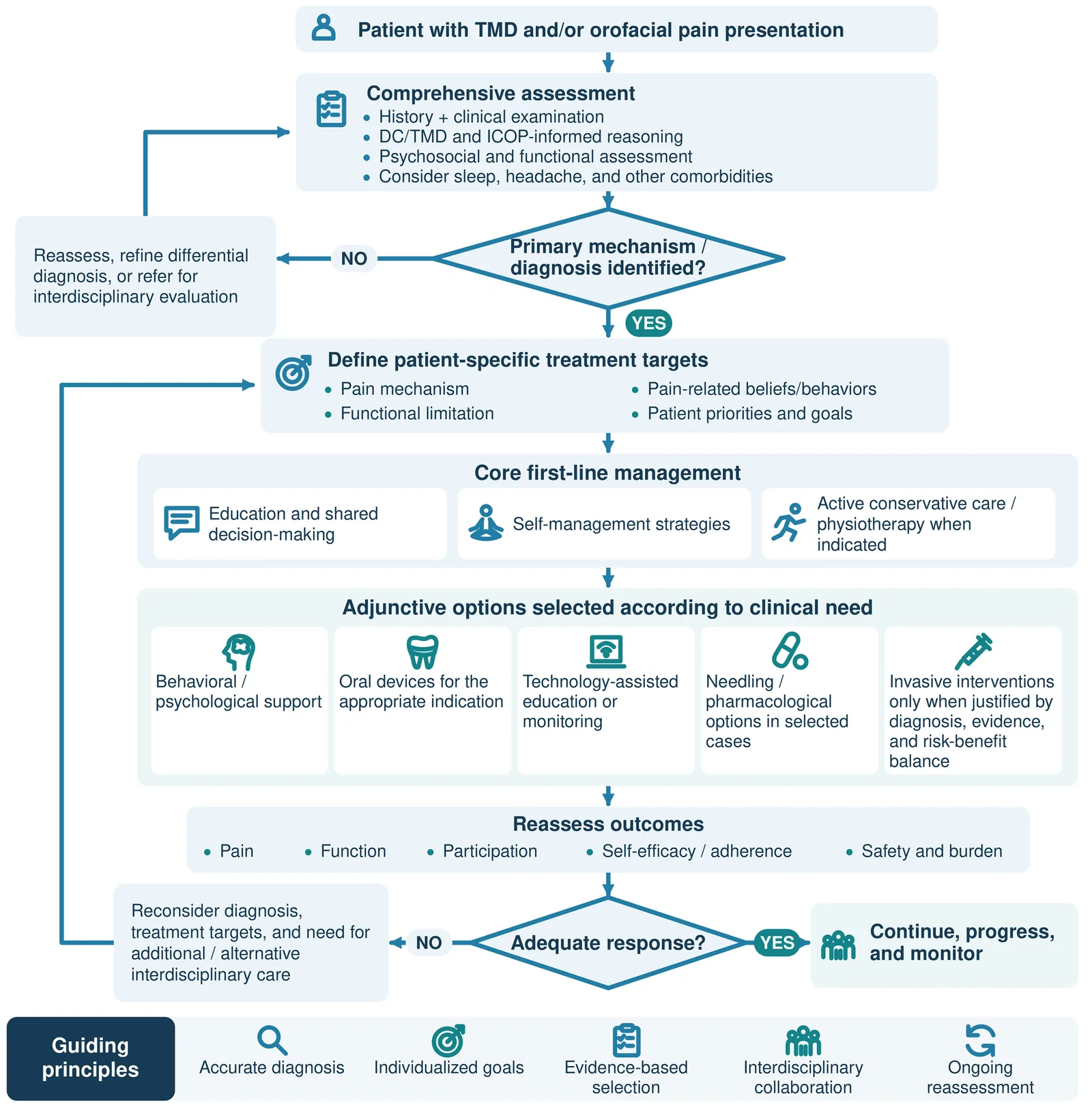
Algorithm for diagnosis-informed, patient-centered multimodal management of TMD and orofacial pain. Assessment and diagnostic reasoning guide patient-specific treatment targets, core first-line management, and adjunctive options selected according to clinical need. Reassessment of pain, function, participation, self-efficacy and adherence, and safety and burden informs continuation or reconsideration of care. The return pathways indicate reassessment when the primary mechanism or diagnosis is uncertain or the clinical response is inadequate. TMD, temporomandibular disorders; DC/TMD, diagnostic criteria for temporomandibular disorders; ICOP, International Classification of Orofacial Pain.

This approach also has implications for professional education and interdisciplinary practice. Clinicians need to recognize the boundaries between TMD and other causes of orofacial pain, communicate uncertainty without overstating diagnosis or treatment effects, and refer when patient needs extend beyond their expertise. Future studies should test not only whether an intervention works on average, but for whom, toward which goal, and with what additional benefit. The promise of multimodal management lies in making these decisions explicit and revisiting them as the patient progresses.
